#  Slow food for lactobacilli: characterization of 1,2-propanediol metabolism in *Levilactobacillus parabrevis* FUA3697 in sourdough

**DOI:** 10.1128/aem.01207-25

**Published:** 2025-08-20

**Authors:** Vi D. Pham, Michael G. Gänzle

**Affiliations:** 1 Department of Agricultural, Food and Nutritional Science, University of Alberta 461698, Edmonton, Alberta, Canada; Universita degli Studi di Napoli Federico II, Portici, Italy

**Keywords:** *Lactobacillus*, silage, cider, starter cultures

## Abstract

Some lactobacilli convert lactate to acetate and 1,2-propanediol in the stationary phase of growth, but the key enzyme of the pathway, lactaldehyde dehydrogenase, has not been characterized in any of the *Lactobacillales*. It was, therefore, the aim of this study to characterize lactate to 1,2-propanediol conversion in *Levilactobacillus parabrevis* FUA3697. The strain converted lactate to 1,2-propanediol during 14 days of incubation. The concentration of 1,2-propanediol was higher in media with maltose, glucose, and fructose as carbon sources when compared to arabinose; the addition of lactate increased the 1,2-propanediol formation. The 1,2-propanediol formation was higher in sourdough than in laboratory media and higher in sorghum sourdoughs than in wheat sourdoughs. The construction of a mutant strain *Lv. parabrevis* FUA3697Δ*aldA* confirmed that AldA is responsible for the conversion, as the mutant strain did not produce 1,2-propanediol. Competition experiments demonstrated that the disruption of *aldA* slightly decreased the competitiveness in wheat sourdoughs but not in sorghum sourdoughs. After incubation of the strains over 28 days at 4°C, the wild-type strain resumed growth and acidification faster than the *aldA*-deficient isogenic mutant. In summary, lactate conversion to acetate and 1,2-propanediol maintains slow metabolic activity over weeks or even months, thus increasing the ecological fitness.

**IMPORTANCE:**

The genes coding for lactate conversion to 1,2-propanediol are relatively broadly distributed in heterofermentative lactobacilli and also present in few homofermentative lactobacilli. To date, the conversion has been verified experimentally only in *Lentilactobacillus buchneri*, few other lentilactobacilli and, more recently, *Levilactobacillus lettrarii*. 1,2-Propanediol is a substrate for further conversion to propionic acid. The metabolic pathway contributes to spoilage of pickles and cider but is considered a beneficial metabolic trait in silage and sourdough fermentations. Our results document that lactobacilli using this pathway can be employed for improved activity and competitiveness of starter cultures that are distributed under refrigerated conditions.

## INTRODUCTION

Most lactobacilli (=*Lactobacillaceae*) emphasize rapid growth by making inefficient use of abundant carbohydrates. Homofermentative lactobacilli convert hexoses by glycolysis to yield lactate as the main product of metabolism, while heterofermentative lactobacilli convert hexoses via the phosphoketolase pathway (1). Both pathways maximize the growth rate instead of maximizing the ATP yield and displace competitors by rapid production of lactic acid (2, 3). Homo- and heterofermentative lactobacilli also operate additional metabolic pathways that increase the ATP yield from hexoses. Homofermentative lactobacilli, with the exception of the *Lactobacillus* clade consisting of the genera *Lactobacillus*, *Amylolactobacillus*, and *Holzapfeliella*, convert pyruvate via pyruvate-formate lyase (4), which is repressed by glucose and inhibited by a low pH. Many heterofermentative lactobacilli generate additional ATP by formation of 1,2-propanediol from lactate during the stationary phase of growth (1).

Bacteria produce 1,2-propanediol by three different pathways, namely, the fermentation of deoxyhexoses, the conversion of methylglyoxal, and the conversion of lactate (5). The deoxyhexose and methylglyoxal pathways have been reported in many bacterial species, including *Escherichia coli*,* Salmonella enterica*,* Lacticaseibacillus rhamnosus*, and *Loigolactobacillus coryneformis*. The formation of 1,2-propanediol from lactate has only been documented in a few genera of the *Lactobacillaceae* (5–8), with the most reports on *Lentilactobacillus buchneri* (9–13).

Lactate is metabolized via an oxidizing branch, which produces ATP and acetate, and a reducing branch that forms 1,2-propanediol (1). 1,2-Propanediol synthesis from lactate is anaerobic and independent of electron acceptors (1, 10). Lactate conversion improves stationary-phase survival of lactobacilli by maintaining the environmental pH, as 1,2-propanediol is not an organic acid, and acetate has a higher pKa than lactate (12). Accordingly, lactate-converting lentilactobacilli occur in long-term food or feed fermentations, such as silage and pickles (9, 10, 14). Lactate conversion also occurs, albeit infrequently, in experimental and commercial sourdough fermentations (13, 15, 16). Genes coding for lactate conversion to 1,2-propanediol are rarely identified in homofermentative lactobacilli but are present in multiple strains of the heterofermentative genera *Limosilactobacillus*, *Secundilactobacillus*, and *Levilactobacillus* (8); however, the predicted phenotype has not been validated. Moreover, whether the pathway contributes to the bacterial ecological fitness has not been documented experimentally.

Genome sequencing of the sourdough isolate *Levilactobacillus parabrevis* FUA3697 revealed that this strain encodes for *aldA* (17). The lactaldehyde dehydrogenase AldA acts on the first step of the reducing branch of lactate metabolism (1). The strain was isolated from an exceptional sourdough that has been back-slopped with 7-day fermentation cycles at less than 8°C (17). The same sourdough also harbors *Levilactobacillus lettrarii*, which converts lactate via 1,2-propanediol to propionate (16). It was the aim of this study to confirm the role of lactaldehyde dehydrogenase in the conversion of lactate to 1,2-propanediol using a mutant derivative with a deleted *aldA* to determine the contribution of AldA to the competitiveness of *Lv. parabrevis* in sourdough fermentations and examine the role of lactate conversion in sourdough reactivation during long-term storage.

## MATERIALS AND METHODS

### Bacterial strains and growth conditions

*Lv. parabrevis* FUA3697 isolated from sourdough (GenBank accession number CP168701) (17) and its isogenic mutant were grown in modified MRS6 (mMRS6) (18) or in commercial MRS media (BD Difco, Sparks, MD, USA) without agitation at 30°C unless otherwise specified. Strains were streaked from glycerol stock stored at −80°C onto said agar and subcultured twice in broth prior to the following experiments. *Escherichia coli* EC1000 was grown aerobically at 37°C in Luria-Bertani (LB) media (BD Difco, Sparks, MD, USA) with the addition of 300 mg/L of erythromycin.

### Construction of isogenic mutant lacking *aldA* coding for lactaldehyde dehydrogenase

The deletion of *aldA* coding for lactaldehyde dehydrogenase from *Lv. parabrevis* FUA3697 was performed using homologous recombination, as described (19) with modifications. The upstream and downstream flanking regions (about 800–1,000 bp) of *aldA* and the plasmid pVPL3002 (20) were amplified by PCR with primers shown in Table 1. The amplicons were purified using the GeneJET PCR Purification Kit (Thermo Fisher, Madison, WI, USA) and digested with DpnI (New England Biolabs, MA, USA) to remove methylated sites. The plasmid containing upstream and downstream regions was constructed using Gibson assembly (New England Biolabs) and transformed into *E. coli* EC1000 by electroporation. Competent cells of *Lv. parabrevis* FUA3697 were prepared by cultivating a subculture in 50 mL of MRS supplemented with 1% glycine to an OD_600nm_ of about 0.5. All subsequent steps were performed at 0–4°C. Cells were washed twice with an equal volume of 10 mmol of MgCl_2_ solution, once with 0.5 M sucrose in 10% glycerol, and resuspended in 0.4 mL of the same solution. About 3 µg of plasmid was mixed with 50 µL of cell suspension, which was transferred to a 0.2 cm cuvette. The mixture was electroporated at 2.5 kV, 400 Ω, and 25 µF and recovered anaerobically in MRS supplemented with 80 mmol of MgCl_2_ at 30°C for 4 h. The cells were then plated onto MRS agar with 1 mg/L of erythromycin and incubated anaerobically for 3 days. Transformants were later grown on MRS agar with 4 mg/L of erythromycin, and single cross-over colonies were identified by PCR. To select for mutants that had lost the plasmid and the *aldA* gene via double crossover, colonies were replica-plated onto MRS and MRS with 4 mg/L of erythromycin. Erythromycin-sensitive colonies were confirmed by PCR and Sanger sequencing. The genome of the mutant strain was sequenced on Oxford Nanopore MinION (Oxford Nanopore Technologies, Oxford, UK), and the number of SNPs relative to the wild type was identified as described (17).

**TABLE 1:** Primers used for construction of the mutant *Lv. parabrevis* FUA3697∆*aldA^a^*

Primer	Description	Sequence (5′→3′)
oVPL 188 F	Amplifies pVPL3002 backbone (20)	ATCCTCTAGAGTCGACCTGC
oVPL 187 R		TACCGAGCTCGAATTCACTGG
oVPL 97 F	Amplifies flanking region inserts in the plasmid (20)	CCCCCATTAAGTGCCGAGTGC
oVPL 49 R		ACAATTTCACACAGGAAACAGC
u/s F	Amplifies upstream flanking region of *aldA* in *Lv. parabrevis* FUA3697	CAGTGAATTCGAGCTCGGTATCATACGGAAGGCTTAGC
u/s R		GTCGCATTGAAACATTATCAATCATAGCGTCG
d/s F	Amplifies downstream flanking region *aldA* in *Lv. parabrevis* FUA3697	GTCGCATTGAAACATTATCAATCATAGCGTCGAAAAAAG
d/s R		CCTTAAAGCCAAAGCCTGATCCTCTAGAGTCGACCTGC
SCO u/s F	Screen for SCO at upstream integration	CGGAACAGCTTCATACGGAAGGCTTAGC
SCO u/s R		CACCTTAAAGCCAAAGCCTG
SCO d/s F	Screen for SCO at downstream integration	TCATACGGAAGGCTTAGCGG
SCO d/s R		CCTTAAAGCCAAAGCCTGGTGACACGAT
DCO F	Screen for DCO of *Lv. parabrevis* FUA3697∆*aldA*	CTTCAGGCGGGTGTGTTTC
DCO R		ACCGGGGTTGCTAGTGTC

^*a*^ F, forward; R, reverse; SCO, single crossover; DCO, double crossover.

### Characterization of metabolites of *Lv. parabrevis* in mMRS6

Cultures of *Lv. parabrevis* FUA3697 and FUA3697∆*aldA* were inoculated with 2% inoculum into fresh mMRS6, providing 10 g/L maltose and 5 g/L each of glucose and fructose as carbon sources or into mMRS with 10 g/L arabinose and 2 g/L of glucose. Both media were used with or without addition of 50 mmol/L lactate and incubated at 30°C for 14 days. Samples containing only the media without cells were used as blank control. Cells were removed by centrifugation, and the supernatant was mixed with 7% perchloric acid in a 1:1 ratio and incubated at 4°C overnight to precipitate proteins. Solids were removed by centrifugation twice for 10 min at 17,000 rcf. The metabolites were separated on an Aminex HPX-87H column (300 × 7.8 mm, 9 µm; Bio-Rad Laboratories, Inc., Redmond, WA, USA) using an Agilent 1200 Series HPLC System (Agilent Technologies, Santa Clara, CA, USA) and quantified with RI and UV_210 nm_ detection as described (21).

### Competition experiment with *Lv. parabrevis* FUA3697 and FUA3697∆*aldA*

Competition experiments were conducted in white wheat or brown sorghum sourdoughs. Separate cultures of the two strains were washed once with an equal volume of sterile tap water and resuspended in the same volume of sterile tap water. The cell density of the cultures was adjusted by dilution to an OD_600nm_ of 0.3. Sourdoughs were inoculated with both strains to a cell count of about 10^8^ CFU/g and fermented for 14 days at 10°C. Three independent replications of wheat and sorghum competition sourdoughs were performed.

In a second experiment, wheat and sorghum sourdoughs were inoculated similarly and back-slopped 10 times with fermentation cycles of 7 days at 15°C. Each fermentation cycle was initiated by mixing 0.4 g of sourdough from the previous fermentation cycle with 2 mL of sterile tap water and 1.8 g of fresh flour (wheat or sorghum). Three independent replications of wheat and sorghum competition sourdoughs were performed.

### Isolation of sourdough DNA

About 2 g of sourdough was homogenized with 25 mL of 0.9% NaCl_2_ solution (w/v). The mixture was centrifuged at 700 (wheat sourdough) or 300 rcf (sorghum sourdough) for 5 min to remove solids. Bacterial cells were harvested from the supernatant by centrifugation, and community DNA was extracted using the DNeasy Blood and Tissue Kit (Qiagen, Hilden, Germany). The quantity and quality of DNA were monitored using a NanoDrop One spectrophotometer (Thermo Fisher, Madison, WI, USA).

### Quantification of gene copies in competition sourdoughs using digital quantitative PCR

To quantify gene copies of *Lv. parabrevis* FUA3697 and its mutant, primers and probes (Table 2) were designed to target the sequence of the deleted gene and the flanking region. Probes were supplied by Integrated DNA Technologies, Inc. (IDT, Coralville, IA, USA), where SUN dye, an equivalent of VIC dye, was used.

**TABLE 2:** Primers and probes used for digital PCR

Primer^*a*^	Description	Sequence (5′→3′)
WT F	Primers and probe (FAM dye) targeting the deleted sequence of *aldA*	CGGGGATGAACTTTCCAAGAAC
WT R		GACATCGATTTGGCCGTTCA
WT probe		/56-FAM/TGGCTGCTG/ZEN/CTGCCACCCACATGGCCAA/3IABkFQ/
MT F	Primers and probe (VIC dye) targeting the flanking region	ATCGCACCTACCGATTTGGA
MT R		ATTGTAAACGCCGGTATGACG
MT probe		/5SUN/GAACTCTGT/ZEN/CGCATTGAAACATTATCAA/3IABkFQ/^*b*^

^*a*^ F, forward; R, reverse.

^*b*^ SUN dye is an equivalent of VIC dye supplied by IDT.

The QuantStudio 3D Digital PCR System used for analysis included a ProFlex 2× flat PCR, 20K chip v2, a chip loader, and a chip reader (Applied Biosystems, Thermo Fisher Scientific). To prepare for digital PCR, the extracted DNA was diluted to 1 mg/L. One multiplex reaction includes the following reagents (final concentration): digital PCR Master Mix v2 (1×), WT and MT primers (0.6 µmol each), probes (0.2 µmol each), DNA template (67 pg), and water to a final volume of 15 µL. The thermal cycler was programmed for chips with three stages: initial denaturation and activation of polymerase (96°C, 10 min), 39 cycles of annealing and extension (58°C, 2 min; and 98°C, 30 s), and final extension (60°C, 2 min). The wild type was quantified using the FAM and VIC fluorescence, and the mutant strain was quantified using the VIC fluorescence. The absolute quantification of gene copies was corrected using Poisson distribution implemented in the QuantStudio 3D AnalysisSuite Cloud Software. Data are shown as mean and standard deviation of log 10 of ratio of gene copies of WT over MT.

### Quantification of metabolites in competition sourdoughs using HPLC

To quantify organic acids and sugars in competition sourdoughs, 1 g of sourdough was homogenized with 9 mL of sterile water. The supernatant was collected by centrifugation and analyzed by HPLC as described above.

### Assessment of acidification of *Lv. parabrevis* FUA3697 and FUA3697∆*aldA* in media

To determine the metabolic activity of stationary-phase cells of *Lv. parabrevis* FUA3697 and its mutant, an acidification assay using bromocresol purple as an indicator dye was performed (22). Cells were streaked from glycerol stock onto mMRS6 agar, incubated anaerobically for 2 days at 30°C, and subsequently stored for 28 days at 4°C. Every 7 days from the first day of storage, several colonies of *Lv. parabrevis* FUA3697 and FUA3697∆*aldA* were suspended in mMRS6 broth and diluted to an OD_600nm_ of 0.3. Of this cell suspension, 200 µL was mixed with 5 µL of bromocresol purple (0.5 g/L) and incubated in a 96-well microtiter plate (Corning, NY, USA). The plate included negative controls (i.e., broth without cell suspension and without dye, broth without cell suspension but with dye and calibrations, and cell suspensions without dye). The microtiter plate was incubated at ambient temperature, and the absorbance at 430 nm was measured every 5 min by a spectrophotometer (Varioskan Flash, Thermo Fisher). The linear relationship between the absorbance of the dye and the pH was determined as described (22). To determine the correlation between pH and the absorbance of the dye, at absorbance of 1.2, 1.39, 1.4, 1.5, 1.6, 1.7, and 1.8 nm, 200 µL of culture without the dye was collected and mixed with 1.8 mL of ultra-filtered 18 MΩ water for pH measurements. Three independent replicates of acidification assay experiments were performed.

## RESULTS

### Phenotypic confirmation and characterization of lactaldehyde dehydrogenase of *Lv. parabrevis* FUA3697 and FUA3697∆*aldA*

Formation of 1,2-propanediol from lactate requires lactaldehyde dehydrogenase as the first enzyme of the pathway converting lactate to 1,2-propanediol (1). The lactaldehyde dehydrogenase encoded by *aldA* was deleted in *Lv. parabrevis* FUA3697∆*aldA* by a double-crossover protocol that leaves no foreign DNA in the genome of the mutant strain (20). The comparison of the genome sequences of *Lv. parabrevis* FUA3697 and FUA3697∆*aldA* (17) showed no deleted region other than the intended *aldA* gene but revealed that the two genomes differed by 59 SNPs. To confirm the phenotype of *Lv. parabrevis* FUA3697 and FUA3697*∆aldA*, the strains were grown in mMRS6 media, and the metabolites were quantified (Table 3). After 14 days of incubation, 1,2-propanediol was detected in cultures of *Lv. parabrevis* FUA3697 but not in cultures of FUA3697∆*aldA* (Table 3).

**TABLE 3:** Metabolism of lactate and production of 1,2-propanediol (1,2 POH) of *Lv. parabrevis* FUA3697 and its isogenic mutant FUA3697∆*aldA* in mMRS6 media with different carbon sources and with or without lactate after 14 days of incubation at 30°C^*a*^

Strain	Substrate						Metabolites (mmol/L)					
	Maltose 10 g/L	Glucose 5 g/L	Fructose 5 g/L	Lactate 50 mmol/L	Maltose	Glucose	Fructose	Mannitol	Lactate	Acetate	Ethanol	1,2 POH
FUA 3697	+	+	+	−	−25.4 ± 0.4	−26.1 ± 1.5	−29.3 ± 0.6	21.1 ± 0.9	83.8 ± 1.5	18.3 ± 1.2	45.6 ± 0.4	2.4 ± 0.4
	+	+	+	+	−24.8 ± 0.3	−26.0 ± 0.6	−27.9 ± 0.2	22.5 ± 0.1	73.0 ± 4.1	27.2 ± 3.1	35.1 ± 2.8	7.6 ± 2.2
FUA| 3697 ∆*aldA*	+	+	+	−	−25.3 ± 0.4	−25.5 ± 1.8	−29.4 ± 0.5	21.0 ± 1.0	85.6 ± 2.0	17.4 ± 0.2	45.1 ± 0.1	n.d.^a^
	+	+	+	+	−24.5 ± 0.5	−23.5 ± 2.7	−27.8 ± 0.0	23.3 ± 1.7	84.1 ± 6.9	19.5 ± 0.6	38.4 ± 2.0	n.d.

	Arabinose 10 g/L	Glucose 2 g/L	Lactate 50 mmol/L	Glucose	Arabinose	Lactate	Acetate	Ethanol	1,2 POH
FUA 3697	+	+	−	−11.7 ± 0.4	−65.5 ± 1.2	73.0 ± 0.3	68.1 ± 3.1	4.3 ± 0.8	1.3 ± 0.2
	+	+	+	−8.2 ± 1.9	−57.7 ± 3.0	63.7 ± 6.0	59.3 ± 3.3	2.1 ± 0.5	1.2 ± 0.1
FUA 3697 ∆*aldA*	+	+	−	−12.4 ± 0.5	−66.0 ± 0.1	75.5 ± 1.6	68.0 ± 2.8	4.4 ± 0.5	n.d.
	+	+	+	−9.8 ± 1.6	−60.5 ± 2.5	69.8 ± 2.3	57.1 ± 8.0	3.1 ± 1.3	n.d.

^*a*^ Positive numbers indicate the formation during incubation; negative numbers indicate consumption. Data are shown as mean ± standard deviation of three independent replicates. n.d., not detected (i.e., below the detection limit of 1 mmol/kg).

The production of 1,2-propanediol by *Lv. parabrevis* FUA3697 was quantified in media with hexoses or pentoses as carbon source and with or without addition of lactate. Overall, 1,2-propanediol was produced in all cases, ranging from 1.2 ± 0.1 to 7.6 ± 2.2 mmol (Table 3). The addition of lactate increased formation of 1,2-propanediol (Table 3, upper panel). The level of 1,2-propanediol was low when arabinose was provided as substrate, regardless of lactate addition (Table 3, lower panel).

### Ecological fitness and lactate metabolism of wild type and mutant strains in long-term fermented wheat and sorghum sourdoughs

**Fig 1:**
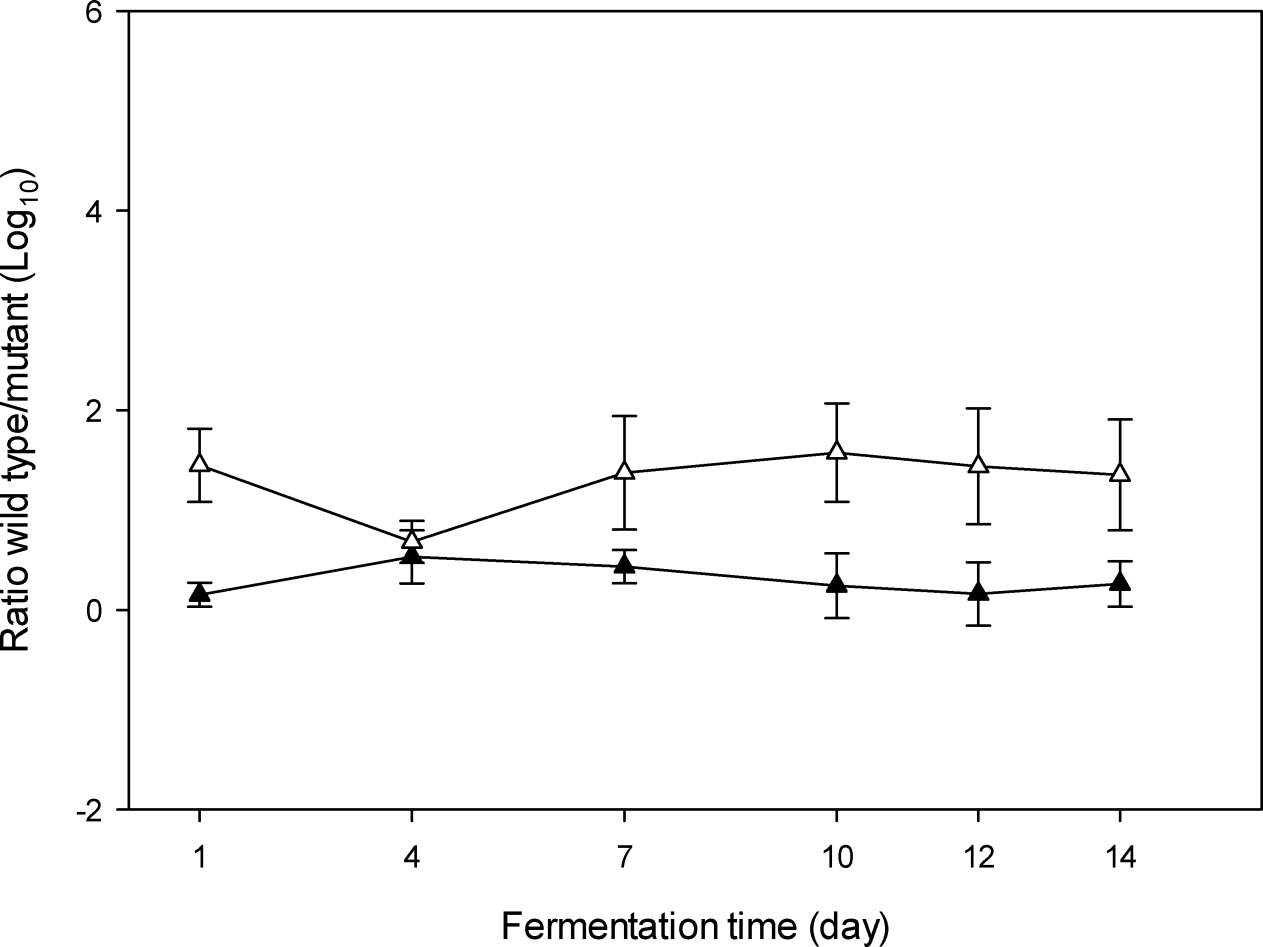
Ratio of the gene copies of *Lv. parabrevis* FUA 3697 and FUA 3697∆*aldA* in wheat (black triangles) and sorghum (white triangles). Sourdough was inoculated with *Lv. parabrevis* FUA 3697 and FUA 3697∆*aldA* and fermented for 14 days at 10°C. The copy number of FUA3697 and FUA3697∆*aldA* was determined using quantitative digital PCR. Data are means ± standard deviation of three independent experiments.

To examine the contribution of the lactate conversion to the ecological fitness of *Lv. parabrevis* FUA3697 and FUA3697∆*aldA* in sourdough, the strains were grown competitively in wheat and sorghum sourdough fermented at 10°C for 14 days. The ratio of gene copy numbers of FUA3697 and FUA3697∆*aldA* was quantified using digital PCR (Fig. 1). In both wheat and sorghum sourdough, the ratio of gene copies remained constant throughout the fermentation period, indicating that cells of the wild type were not more abundant than cells of the mutant strain. Since DNA from dead or metabolically inactive cells remains detectable by qPCR (23), the DNA copy numbers may not accurately reflect the live cell counts of the two strains.

In wheat sourdough, maltose and fructose were consumed gradually while glucose increased (Table 4). In sorghum sourdough, maltose and fructose were no longer detected after 4 days, while glucose was detected at a decreased concentration throughout 14 days (Table 4). The high buffering capacity of sorghum when compared to wheat (24) resulted in a higher concentration of lactate (Table 4). Lactate increased over the first 4 days of fermentation and remained stable afterwards. The higher concentration of lactate in sorghum related to a higher production of 1,2-propanediol. In both wheat and sorghum, 1,2-propanediol and acetate concentrations but not the lactate concentration continuously increased throughout the fermentation, indicating that the wild type remained metabolically active over the 14-day incubation period.

**TABLE 4:** Carbon sources of wheat and sorghum and metabolites (mmol/g sourdough) of the co-culture of *Lv. parabrevis* FUA3697 and FUA3697∆*aldA* in wheat or sorghum sourdough at 10°C during 14 days of fermentation^*a*^

	Lactate	Acetate	1,2 POH	Maltose	Glucose	Fructose^*b*^	Ethanol	Mannitol
Day 1								
Wheat	22.9 ± 4.1	8.59 ± 1.4^a^	5.63 ± 4.8^a^	111 ± 16.5	47.2 ± 20	31.7 ± 0.97	11.3 ± 4.2	0.8 ± 0.2
Sorghum	46.4 ± 5.5	16.6 ± 1.7^A^	8.49 ± 1.4^A^	0.663 ± 1.0	71.1 ± 4.5	19.2 ± 5.5	6.43 ± 6.0	15.0 ± 1.7
Day 4								
Wheat	106 ± 27	24.7 ± 5.0^b^	7.88 ± 0.7^b^	143 ± 25	82.0 ± 33	32.9 ± 8.2	23.2 ± 5.1	11.1 ± 2.1
Sorghum	140 ± 37	46.9 ± 5.2^B^	9.40 ± 0.9^A^	n.d.^*c*^	46.5 ± 19	n.d.	30.4 ± 7.9	28.2 ± 5.1
Day 7								
Wheat	98.1 ± 1.6	25.2 ± 0.39^b^	8.85 ± 0.41^b^	96.5 ± 6.8	70.7 ± 21	19.9 ± 0.93	26.8 ± 7.1	11.7 ± 1.7
Sorghum	158 ± 17	51.8 ± 5.2^B^	16.0 ± 1.4^B^	n.d.	21.3 ± 6.1	n.d.	44.5 ± 8.1	15.2 ± 1.7
Day 10								
Wheat	114 ± 26	30.7 ± 3.6^bc^	10.5 ± 1.7^b^	91.5 ± 22	83.9 ± 31	17.8 ± 5.0	31.9 ± 4.0	13.5 ± 4.6
Sorghum	148 ± 16	65.7 ± 3.6^CD^	21.1 ± 1.8^C^	n.d.	7.10 ± 3.2	n.d.	44.7 ± 19	7.88 ± 2.4
Day 12								
Wheat	116 ± 8.9	32.0 ± 3.1^bc^	11.7 ± 2.1^b^	86.8 ± 18	82.1 ± 22	16.7 ± 2.6	40.2 ± 13	16.0 ± 3.1
Sorghum	201 ± 44	76.4 ± 6.0^D^	29.9 ± 0.34^D^	n.d.	7.24 ± 3.1	n.d.	47.8 ± 19	11.7 ± 8.2
Day 14								
Wheat	117 ± 5.4	34.8 ± 2.6^c^	13.4 ± 2.6^b^	76.5 ± 1.9	84.5 ± 15	15.1 ± 0.88	36.9 ± 1.1	15.7 ± 1.0
Sorghum	167 ± 6.3	75.2 ± 3.8^D^	31.2 ± 2.9^D^	n.d.	2.25 ± 1.0	n.d.	57.3 ± 0.45	3.75 ± 2.0

^*a*^ Data are means ± standard deviation of three independent experiments.

^*b*^ Values include fructose released from fructo-oligosaccharides during sample preparation.

^*c*^ n.d., not detected (i.e., below the detection limit of 1 mmol/kg). Numbers that do not share superscript lowercase letters (wheat) or superscript capitalized letters (sorghum) are significantly different, as determined by one-way ANOVA test, followed by Tukey’s HSD for pairwise comparison. Only acetate and 1,2-propanediol were subjected to statistical analysis.

### Ecological fitness and lactate metabolism of wild type and mutant strains in back-slopped wheat and sorghum sourdoughs

A second experiment was conducted by back-slopping of sourdoughs for 10 fermentation cycles, which were incubated for 7 days each. To limit the total time required for the experiment to 100 days, the fermentation temperature was increased from 10 to 15°C. The wheat or sorghum flour was inoculated with an equal cell count of *Lv. parabrevis* FUA3697 and its mutant FUA3697∆*aldA*, and the resulting sourdoughs were back-slopped with 10% inoculation. The repetitive cycles resulted in a similar profile of carbon sources and metabolites at each time point (Table 5). The production of 1,2-propanediol in back-slopped sorghum sourdough gradually decreased from 23 to 16 mmol/kg over the 10 fermentation cycles with a concomitant decrease in the concentration of acetate (Table 5). In back-slopped wheat sourdough, the production of 1,2-propanediol and acetate remained unchanged over the 10 fermentation cycles.

**TABLE 5:** Carbon sources of wheat and sorghum and metabolites (mmol/g sourdough) of the co-culture of *Lv. parabrevis* FUA3697 and FUA3697∆*aldA* in wheat or sorghum sourdough back-slopped every 7 days for 10 cycles at 15°C^*a*^

	Lactate	Acetate	1,2 POH	Maltose	Glucose	Fructose	Ethanol	Mannitol
Cycle 1								
Wheat	95.1 ± 17	35.9 ± 14	15.0 ± 5.7^a^	41.3 ± 4.6	36.3 ± 22	12.9 ± 0.63	14.5 ± 2.4	5.76 ± 3.5
Sorghum	117 ± 5.1	54.9 ± 7.0^A^	23.0 ± 1.2^A^	n.d.^*b*^	12.1 ± 3.7	n.d.	15.7 ± 5.6	2.32 ± 0.29
Cycle 2								
Wheat	73.6 ± 11	17.5 ± 8.0	11.5 ± 0.16^a^	53.6 ± 8.2	58.5 ± 11	11.9 ± 1.6	7.60 ± 4.7	9.15 ± 1.7
Sorghum	108 ± 5.8	46.3 ± 5.3^AB^	19.1 ± 1.9^A^	n.d.	14.6 ± 0.19	n.d.	13.2 ± 5.0	3.14 ± 0.6
Cycle 4								
Wheat	72.8 ± 6.7	13.9 ± 8.0	11.8 ± 0.83^a^	44.5 ± 7.8	39.6 ± 18	9.93 ± 1.7	10.3 ± 3.0	8.89 ± 0.54
Sorghum	118 ± 2.5	42.4 ± 1.1^B^	20.9 ± 1.2^AB^	n.d.	13.7 ± 1.3	n.d.	16.1 ± 0.67	2.63 ± 0.17
Cycle 6								
Wheat	78.6 ± 3.8	18.8 ± 9.1	11.6 ± 0.18^a^	44.4 ± 2.1	34.0 ± 8.9	11.6 ± 2.2	11.6 ± 3.0	6.06 ± 3.0
Sorghum	124 ± 8.8	37.4 ± 1.9^B^	18.8 ± 2.8^ABC^	n.d.	13.2 ± 0.9	n.d.	16.5 ± 3.1	3.87 ± 1.0
Cycle 8								
Wheat	77.7 ± 5.5	15.8 ± 7.1	11.8 ± 1.1^a^	53.7 ± 3.5	25.7 ± 6.5	13.1 ± 1.8	7.15 ± 0.54	4.21 ± 1.25
Sorghum	122 ± 10	37.3 ± 1.8^B^	16.6 ± 1.2^BC^	n.d.	12.6 ± 1.2	n.d.	15.8 ± 1.8	4.35 ± 1.0
Cycle 10								
Wheat	80.9 ± 3.3	16.3 ± 8.6	11.9 ± 0.84^a^	57.1 ± 5.6	27.2 ± 6.0	12.2 ± 1.7	10.2 ± 2.4	5.01 ± 3.5
Sorghum	123 ± 8.9	41.1 ± 3.9^B^	15.8 ± 1.0^C^	n.d.	13.1 ± 1.0	n.d.	18.9 ± 1.9	4.46 ± 0.75

^*a*^ Metabolites of selected cycles are shown. Data are means ± standard deviation of three independent experiments.

^*b*^ n.d., not detected (i.e., below the detection limit of 1 mmol/kg). Numbers that do not share a superscript lowercase letter (wheat) or a superscript capitalized letter (sorghum) are significantly different, as determined by one-way ANOVA test, followed by Tukey’s HSD for pairwise comparison. Only data for acetate and 1,2-propanediol were subjected to statistical analysis.

**Fig 2:**
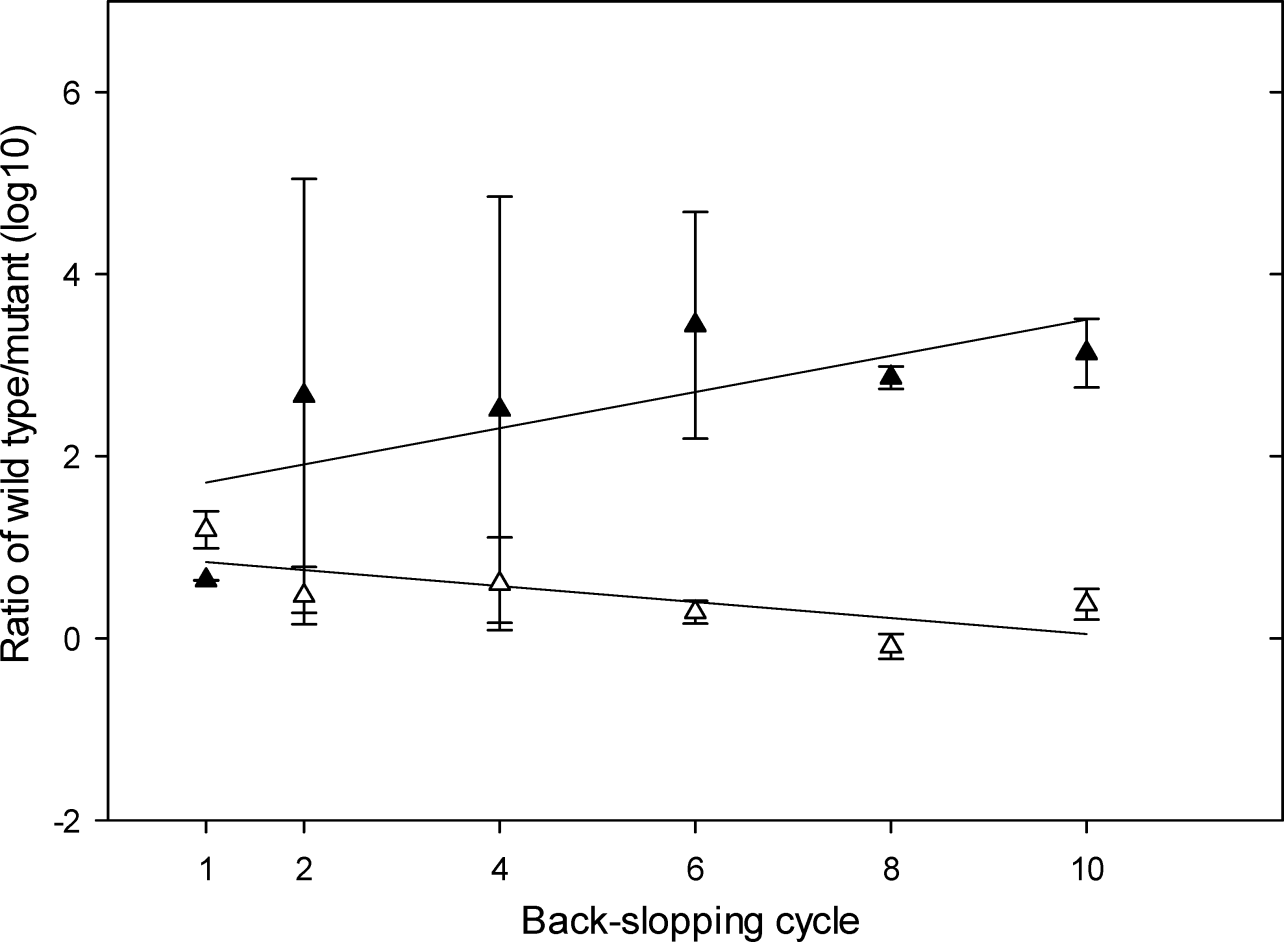
Ratio of the gene copies of *Lv. parabrevis* FUA 3697 and FUA 3697∆*aldA* in wheat (black triangles) and sorghum (white triangles). Sourdough was inoculated with *Lv. parabrevis* FUA 3697 and FUA 3697∆*aldA* fermented for 7 days at 15°C, and then back-slopped with 10% inoculum for 10 additional fermentation cycles. The copy numbers of FUA3697 and FUA3697∆*aldA* were determined using quantitative digital PCR. Data are means ± standard deviation of three independent experiments. Lines indicate regression lines obtained by linear regression.

The competition in back-slopped sourdough between *Lv. parabrevis* FUA3697 and FUA3697∆*aldA* was determined by the log-transformed ratio of gene copy numbers using digital PCR (Fig. 2). Here, DNA from dead or inactive cells does not interfere with an accurate quantification of wild-type and mutant strains because dead cells are diluted out during every back-slopping step. A linear regression was applied, and the slope was used to measure the ecological fitness (21). In back-slopped sorghum sourdough, the mutant was slightly more competitive than the wild type. However, in back-slopped wheat sourdough, the wild type outcompeted the mutant after 10 fermentation cycles (Fig. 2), indicating that back-slopping and type of flour impact the ecological fitness to the strain.

### Lactate metabolism contributes to rapid acidification upon sourdough reactivation after 4-weeks of storage

**Fig 3:**
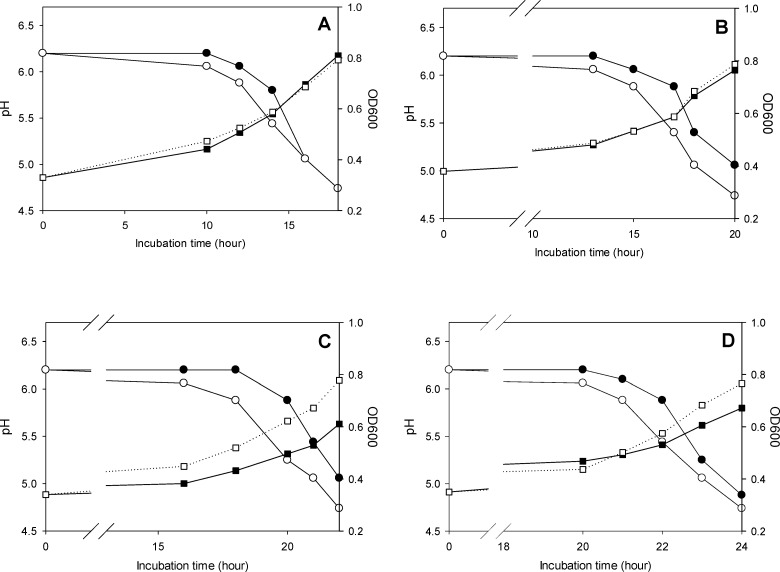
Acidification and growth of *Lv. parabrevis* FUA3697 (white) and its isogenic mutant FUA3697∆*aldA* (black) in mMRS6 media. Acidification was measured by absorbance of bromocresol purple dye at 430 nm and pH (circles). Growth was measured by absorbance of cell density at 600 nm (squares). The strains were stored at 4°C and tested for acidification and growth at 7 (**A**), 14 (**B**), 21 (**C**), and 28 (**D**) days. The *x*-axes are shown with breaks for easy visualization. Data are representative of three independent incubations.

The lactaldehyde dehydrogenase-mediated conversion of lactate to 1,2-propanediol relieves acid stress on bacterial cells in long-term fermentations (12); therefore, the metabolic activities of *Lv. parabrevis* FUA3697 and FUA3697∆*aldA* over 4 weeks of storage were assessed. Strains were incubated at 4°C for 28 days, and cells were collected for the analysis of growth and acidification in mMRS6 every 7 days (Fig. 3). The acidification assay used bromocresol purple as the indicator dye. The analyses were independently replicated thrice, and data are shown as a representative of three replications. Over 28 days of incubation, the wild-type strain consistently acidified faster than the mutant strain (Fig. 3). Growth or biomass does not always correlate to acidification (22), but the wild-type strain also grew faster than the mutant strain (Fig. 3C and D). This result indicates that lactate conversion maintains the metabolic activity of *Lv. parabrevis* FUA3697 during long-term incubation, thus facilitating a rapid re-growth upon transfer to a new medium.

## DISCUSSION

Homologs of lactaldehyde dehydrogenase, the first enzyme in lactate metabolism pathway to 1,2-propanediol*,* have been identified in more than half of the 36 genera of the *Lactobacillaceae* (8). The corresponding phenotype has only been reported in *Lentilactobacillus buchneri*, *Lt. parabuchneri*, *Lt. parafarraginis,* and *Lv. lettrarii* (10, 12, 13, 16, 25). This study characterized the phenotype of lactaldehyde dehydrogenase in *Lv. parabrevis* FUA3697 *in vitro* and in sourdough fermentations. Deletion of the gene *aldA* coding for lactaldehyde dehydrogenase confirmed that this enzyme is required for the formation of 1,2-propanediol. In the metabolism of fucose and rhamnose, lactaldehyde dehydrogenase operates in the reverse direction to oxidize lactaldehyde to lactate as a main product of fucose or rhamnose metabolism (7). Other lactobacilli, including *Lacticaseibacillus rhamnosus*, however, reduce lactaldehyde to 1,2-propanediol (26).

*Lt. buchneri* does not metabolize lactate until cells reach the stationary phase of growth (13). The pH, the high lactic acid concentration, and the NaCl concentration were all shown to influence the catabolism of lactate (10, 12). Higher lactate concentrations increased the conversion to 1,2-propanediol in *Lv. parabrevis* FUA3697 (Tables 3 to 5), which aligned with the result of *Lt. buchneri* (12). Our result additionally showed that the metabolism of pentoses decreased the 1,2-propanediol formation. Acetate is a major product of pentose metabolism but not of hexose metabolism of lactobacilli (Table 3) (1), and high acetate concentrations may inhibit lactate conversion to 1,2-propanediol and acetate. Lactate conversion to acetate and 1,2-propanediol consumes protons, which limits the growth of lactobacilli in sourdough (27) and thus also supports further hexose metabolism and growth.

Lentilactobacilli are occasional but not frequent isolates from sourdough (15). Most sourdoughs are maintained by frequent back-slopping with up to four fermentation cycles per day (28, 29). The sourdough in which *Lv. parabrevis* FUA3697 was identified as one of the most abundant organisms, however, was maintained by fermentation at 4°C with 7-day propagation cycles (17). These exceptional fermentation conditions also selected for the exceptional 1,2-propanediol-producing lactobacilli (16, 17).

The effects of substrates on lactate metabolism were further illustrated in the competition experiment in sourdough, which constitutes an accurate and fairly sensitive tool to determine the ecological fitness of lactobacilli *in situ* and *in vivo* (30, 31). Wheat delivers maltose, while sorghum provides glucose as the major carbon source (32). In wheat sourdoughs, the concentration of 1,2-propanediol was lower than that in sorghum sourdough in two fermentation conditions, corresponding to a low concentration of acetate (Tables 4 and 5). In sorghum sourdoughs, fructose was depleted, but acetate continued to accumulate possibly due to the activity of NADH-dependent 2-ene reductases. This enzyme is active on phenolic acids, which are more abundant in sorghum than in wheat (21, 33).

The availability of electron acceptors in sorghum may explain the equal competition between *Lv. parabrevis* FUA3697 and its mutant (Fig. 2). Substrates, including fructose and phenolic acids, are preferably used as electron acceptors by heterofermentative lactobacilli to recycle NADH and generate ATP in concomitant with formation of acetate (1, 34). Sorghum contains relatively high concentrations of hydroxycinnamic acids (35). With a plentiful source of electron acceptors for growth, the advantage of lactate conversion was likely diminished in sorghum sourdough. This advantage was amplified in wheat where electron acceptors were scarce. During 90 days of ripening of plant-based cheese, where electron acceptors were also available, *L. buchneri* produced only low concentrations of 1,2-propanediol (25).

The antimicrobial activity of hydroxycinnamic acids in wheat and sorghum is unlikely to inhibit growth of *Lv. parabrevis* FUA3697 and its mutant. Both strains possess homologs of the hydroxycinnamic esterase (Lp_0796) and reductase (HcrA) of *Lp. plantarum* WCFS1 with 30 and 51% identity, respectively (36, 37). Phenolic acids exert antimicrobial activity against various species of lactobacilli (38). Growth of *Furfurilactobacillus milii* in sorghum sourdough was not affected by the deletion of genes coding for metabolism (and detoxification) of hydroxycinnamic acids (21).

In silage fermentations and during spoilage of cucumbers, lactobacilli that convert lactate to 1,2-propanediol remain metabolically active over weeks or months of incubation (10, 12). *Lt. buchneri,* commonly found in silage or maize fermentations and a few sourdoughs, is metabolically active as long as the silage is fermented (10, 15).

Industrial and artisanal sourdoughs are maintained in an active state by frequent back-slopping, typically more than once per day (28). Frequent propagations are resource-intensive since they require labor, time, and ingredients (39). To account for holidays or weekends, refrigerated storage of sourdoughs is a common practice (40). Some of the commercial sourdough starter cultures are distributed refrigerated (28). Upon reactivation of refrigerated sourdoughs, however, the sourdough lactobacilli resume growth only after an extended lag phase (41). Therefore, a more rapid acidification upon storage or refrigeration is a desirable trait. Our study demonstrated that during 28 days of storage at 4°C, the wild-type strain *Lv. parabrevis* FUA3697 with lactate metabolism acidified and grew more rapidly in media than its mutant lacking the pathway (Fig. 3). Lactobacilli that metabolize lactate to 1,2-propanediol or propionate are, thus, a suitable choice for sourdough starter cultures that acidify rapidly after extended refrigerated storage.

The metabolism of lactate by *Lv. parabrevis* may contribute to an extended mold-free shelf life of sourdough bread. Lactate catabolism to 1,2-propanediol also accumulates acetate, a compound with stronger antifungal activity than lactate, and supports the formation of propionic acid when 1,2-propanediol-consuming lactobacilli are present (13, 42). The use of acetate and propionate to extend the mold-free shelf life of bread is limited by the impact of these acids on the sensory properties of bread (43). The aerobic stability of silage was also improved when lactobacilli converting lactate to acetate and propionate were present (14, 44, 45).

In conclusion, this study determined the conversion of lactate to acetate and 1,2-propanediol in *Levilactobacillus parabrevis* FUA3697 in media, wheat, and sorghum sourdough, confirming the role of lactaldehyde dehydrogenase encoded by *aldA* in the pathway. The study further demonstrated that upon revival during the 28-day incubation, *Lv. parabrevis* FUA3697 acidified the media faster than its mutant lacking the pathway. Lactobacilli generally approach carbohydrate conversion by metabolic pathways that prioritize fast growth rates and rapid but inefficient conversion. In contrast, production and conversion of 1,2-propanediol support slow but sustained growth and metabolism in the stationary phase of growth, making 1,2-propanediol one of the pathways that support the “slow food” lifestyle of lactobacilli. Lactobacilli using this pathway can be employed for improved activity and competitiveness of starter cultures and for improved food and feed quality.
